# The Effect of Kitchen Ventilation Modification on Independent and Combined Associations of Cooking Fuel Type and Cooking Duration with Suicidal Ideation: A Cross-Sectional Study

**DOI:** 10.3390/toxics10120721

**Published:** 2022-11-24

**Authors:** Caiyun Zhang, Ning Kang, Xiaoyu Hou, Gaohua Chang, Yinghao Yuchi, Xiaotian Liu, Wenqian Huo, Zhenxing Mao, Jian Hou, Chongjian Wang

**Affiliations:** 1 Department of Epidemiology and Biostatistics, College of Public Health, Zhengzhou University, Zhengzhou 450001, China; 2 Department of Occupational and Environmental Health Sciences, College of Public Health, Zhengzhou University, Zhengzhou 450001, China

**Keywords:** solid fuel use, cooking duration, kitchen ventilation condition, suicidal ideation, rural

## Abstract

Although household air pollution (HAP) is associated with an increased risk of mental disorders, evidence remains scarce for the relationship between HAP and suicidal ideation. A total of 21,381 qualified participants were enrolled on the Henan Rural Cohort Study. HAP information including cooking fuel type, cooking duration and kitchen ventilation was collected by questionnaires. Suicidal ideation was evaluated by item nine of the Patient Health Questionnaire-9 (PHQ-9). Independent and combined associations of cooking fuel type and cooking duration with suicidal ideation were explored by logistic regression models. Analyses were conducted in different kitchen ventilation groups to detect the potential effect modification. The adjusted odds ratio (OR) and 95% confidence interval (95% CI) of solid fuel users versus clean fuel users for suicidal ideation was 1.37 (1.16, 1.62), and the risk of suicidal ideation increased by 15% (95% CI: 5%, 26%) for each additional hour of the cooking duration. Participants cooking with solid fuel for long durations were related to the highest risk of suicidal ideation (OR (95% CI): 1.51 (1.22, 1.87)). However, all these associations were not observed in those cooking with mechanical ventilation. Mechanical ventilation ameliorated relationships between solid fuel use and long-duration cooking with suicidal ideation.

## 1. Introduction

Accounting for about 15% of all deaths, suicide is a public health issue of global proportions and imposed enormous psychological, physiology, and economic burden at the individual, social, and national levels [1]. In 2019, the World Health Organization (WHO) reported 703,000 people died by suicide worldwide, and 77% of them took place in low- and middle-income countries [2]. This problem will become even more pronounced when the first direct precursor to suicide, namely suicidal ideation, is considered. It is estimated that approximately 160 million persons express suicidal ideation each year [3]. Since suicide and suicidal ideation are multifactorial, with biological, psychological, clinical, social, cultural and environmental causes [4], identifying modifiable risk factors for suicidal ideation and addressing them through interventions at the population level is of paramount importance for scientific research. In addition to the conventional influencing factors such as stress and insufficient social support [5,6,7], recent studies reported a consistently positive correlation between ambient air pollutants and suicidal ideation [8,9]. However, people spend most of their time indoors [10], and no research has examined the relationship between household air pollution (HAP) with suicidal ideation.

HAP has imposed a significant health burden on people worldwide and become the 10th largest contributor to disability-adjusted life years (DALYs) [11]. The incomplete combustion of solid fuels is a principal contributor to HAP, especially in developing countries where solid fuels are the primary fuel [12,13]. According to the WHO, roughly 2.6 billion people still cook by using solid fuels, which has led to more than 3.8 million people dying prematurely in 2021 [14]. When combusted indoors, solid fuels can produce a high level of HAP such as fine particulate matter (PM_2.5_) [15]. As reported previously, PM_2.5_ inhalation from HAP caused by incomplete combustion of solid fuels accounts for the bulk of the total PM_2.5_ exposure among the Chinese rural population, with 68% attributed to the residential sector [16]. Previous studies have reported that PM_2.5_ could induce inflammation and oxidative stress which may be a potential mechanism in the development of suicidal ideation [17,18,19]. Thus, there is an emerging and pressing need to investigate the relationship between HAP and suicidal ideation in rural areas.

A growing number of studies have investigated the positive association between solid fuel use and mental health problems such as depression and anxiety [20,21]. However, no existing research has estimated the role of solid fuel use on suicidal ideation. Furthermore, residential exposure to HAP-related PM_2.5_ concentrations is also influenced by a variety of factors, such as the type of domestic cooking fuel used in the kitchen, the frequency and duration of time spent in cooking, as well as the kitchen ventilation condition [22,23,24]. Most studies have only focused on the harmful impact of solid fuels and ignored the effects of cooking duration and ventilation.

Thus, in this research, we focused on the type of domestic cooking fuels, the duration of cooking activities, and kitchen ventilation conditions, which are of great significance in predicting HAP and hypothesized that mechanical ventilation instruments used in the kitchen could ameliorate the relationships of domestic solid fuel using and long-duration daily cooking with suicidal ideation. Therefore, this study aimed to (1) investigate the relationships between domestic cooking fuel type and daily duration of cooking with suicidal ideation; (2) examine the effect of kitchen ventilation conditions on these relationships.

## 2. Materials

### 2.1. Study Population

Participants of this cross-sectional research were drawn from the Henan Rural Cohort Study, which was performed in five rural areas (Xinxiang, Tongxu, Yuzhou, Suiping, and Yima counties) of Henan province via multistage stratified cluster sampling. A total of 41,893 people were invited between July 2015 and September 2017, of which 39,259 participants aged 18 to 79 years ended up participating in the study for a response rate of 93.7%. A detailed description of this cohort can be found elsewhere [25]. The exclusion criteria of this research included (1) participants who did not complete the cooking questionnaires (*n* = 9244); (2) missing fuel type (*n* = 48), ventilation (*n* = 18) and cooking duration (*n* = 16) information; (3) who did not cook regularly (weekly or more frequently) (*n* = 8533); (4) missing information on suicidal ideation (*n* = 19). Ultimately, 21,381 participants were included in this research. The sample size of this study was calculated to be adequate and the detailed calculations are shown in the Supplementary Material. All individuals offered written informed consent, and researchers acted in compliance with the guidelines promulgated by the Declaration of Helsinki.

### 2.2. Exposure Assessment

Self-reported questionnaires were used to obtain HAP exposure information regarding the domestic type of cooking fuel, daily duration of cooking activity and kitchen ventilation condition [26,27]. Initially, participants were asked whether they cooked at home regularly in the past year. For participants who reported cooking regularly, the following three questions were asked further. First, “Which of the following fuels is most commonly used for cooking?”; secondly, “How often do you usually cook each week?” and “How long each meal takes?”; and finally, “What is the main method of ventilation used when cooking at home?”. Participants who reported using electricity, natural gas or liquid gas for cooking were considered clean fuel users, while those who used coal or wood were categorized as solid fuel users. The average daily duration of cooking is calculated as the cooking duration for each meal multiplied by the weekly number of cooking activities divided by seven. According to the median, daily cooking duration is further dichotomized into long duration (≥1.5 h/day) and short duration (<1.5 h/day). Kitchen ventilation condition is divided into natural ventilation and mechanical ventilation (with range hood or extractor).

### 2.3. Outcome Assessment

In line with multiple previous studies [28,29], item nine of the Patient Health Questionnaire-9 (PHQ-9) was performed to evaluate suicidal ideation. The specificity and sensitivity of this approach in identifying suicide risks of individuals in primary care practice have been confirmed [30]. Suicidal ideation was collected by asking the participants “How many times in the last two weeks did you get haunted by the thought that you were better off dead or hurting yourself”. Four levels of answers were provided: 1—Not at all, 2—A few days, 3—Over half of the time, 4—Almost on a daily basis. Only participants who reported “not at all” were assigned to the non-suicidal ideation group. Participants who selected the remaining three options were allocated to the suicidal ideation group.

### 2.4. Covariates Estimate

Covariates information were identified as potential confounding factors between HAP and suicidal ideation and was captured by trained staff through structured questionnaires, including socio-demographic characteristic (age, gender, educational level, marital status, and average monthly income), lifestyle factors (smoking status, drinking status, fruit and vegetable consumption, high-fat diet and physical activity) as well as a personal history of chronic diseases (hypertension, coronary heart diseases (CHD), stroke and type 2 diabetes mellitus (T2DM)). Educational level was divided into elementary school or below, junior high school and senior high school or above. Marital status was divided into two levels: married/cohabiting or widowed/divorced/separated/single. Average monthly income was classified into <500 RMB, 500–1000 RMB or ≥1000 RMB. Smoking status and drinking status were categorised into three levels: never, former and current. Physical activity level was estimated in line with the International Physical Activity Questionnaire and was further grouped into low, moderate and high. Sufficient vegetable and fruit intake were regarded as more than 500 g of vegetable and fruit consumption per day. High-fat diets were defined as consuming exceeding 75 g of meat (livestock and poultry) each day. The individual history of hypertension, CHD, stroke and T2DM were collected through laboratory tests, physical examination or self-reported clinician’s diagnosis. According to standardized protocols, participants’ weight and height were measured twice by trained staff, and the mean value was calculated for further analysis. Body mass index (BMI) is equivalent to weight divided by height square.

### 2.5. Statistical Analysis

The basic characteristics of included participants were presented across suicidal ideation status. Descriptive analyses of continuous variables were stated as mean ± standard deviation (SD), and categorical variables were displayed as frequency and proportions. Student’s *t*-test and Chi-squared test were utilized to compare differences of continuous and categorical variables between the suicidal ideation and non-suicidal ideation group, respectively.

Binary logistic regression models were performed to explore the relationships between domestic cooking fuel type and the daily duration of cooking activity with suicidal ideation. Models were adjusted by age, gender, region, educational level, marital status, average monthly income, smoking and drinking status, high fat diet, fruit and vegetable intake, physical activity, BMI, hypertension, CHD, stroke, T2DM, fuel type, cooking duration and ventilation. In addition, the combined relationships between domestic cooking fuel type and dichotomous duration of cooking were also examined. To test the stability of these associations, all analyses were conducted across gender. Furthermore, the effect of kitchen ventilation conditions on these relationships was also examined. 

Data analysis was carried out using the Statistical Package for the Social Sciences version 21.0 (IBM-SPSS Inc., Armonk, NY, USA) and R software version 4.0.3. Two-tailed *p* values < 0.05 were deemed to be statistically significant.

## 3. Results

### 3.1. Basic Characteristics of Participants

Table 1 describes the characteristics of participants according to suicidal ideation status. A total of 21,381 participants aged 55.37 ± 11.83 years were enrolled in this research, including 4872 (22.79%) men and 16,509 (77.21%) women. Among them, 824 participants were identified with suicidal ideation with a prevalence rate of 3.85%. Compared with the ones without suicidal ideation, participants with suicidal ideation tended to be older, women, widowed/divorced/separated/single, have a lower level of social-economic status (educational level and average monthly income), have more proportion of never smoked and never drank, intake less fat and more fruit and vegetable, have lower BMI and higher prevalence rate of chronic diseases (stroke, CHD and T2DM) (All *p* < 0.05). Additionally, participants with suicidal ideation were more prone to cook with solid fuel, longer duration and less extensive use of mechanical ventilation equipment in the kitchen (All *p* < 0.05), relative to non-suicidal ideation participants.

### 3.2. Independent and Combined Associations of Domestic Cooking Fuel Type and Cooking Duration with Suicidal Ideation

Table 2 displays the independent relationships between domestic cooking fuel type and cooking duration with suicidal ideation. In the unadjusted model, the estimated odds ratios (ORs) and 95% confidence intervals (95% CIs) of the solid fuel use group versus clean fuel use group and each 1 h increase in daily cooking duration were 1.88 (1.62, 2.19) and 1.25 (1.16, 1.36), respectively, among the total population. After adjusting for age, gender, region, educational level, marital status, average monthly income, smoking status, drinking status, high fat diet, fruit and vegetable intake, physical activity, BMI, hypertension, CHD, stroke, T2DM, ventilation and fuel type or cooking duration, these positive associations were still significant. The ORs (95% CI) of solid fuel users versus clean fuel users and each 1 h increase in daily cooking duration for suicidal ideation were 1.37 (1.16, 1.62) and 1.15 (1.05, 1.26) in the multivariable-adjusted models, respectively. In stratified analyses, the adjusted ORs (95% CIs) of solid fuel users versus clean fuel users and each 1 h increase in cooking duration for suicidal ideation were 1.30 (0.84, 1.99) and 1.26 (1.00, 1.56) in men, and 1.39 (1.16, 1.67) and 1.14 (1.03, 1.26) in women, respectively.

**Figure 1:**
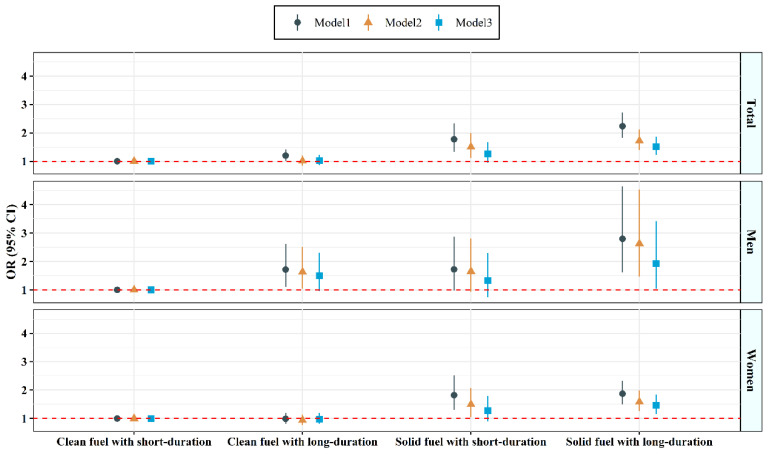
Combined associations of fuel type and cooking duration with suicidal ideation. Model 1 was unadjusted; Model 2 adjusted for age and region in men and women, and additionally adjusted for gender in total; Model 3 further adjusted for marital status, educational level, average monthly income, smoking status, drinking status, high fat diet, fruit and vegetable intake, physical activity, BMI, hypertension, T2DM, CHD, and stroke, ventilation based on model 2.

Figure 1 illustrates the combined relationships between domestic cooking fuel type and dichotomous cooking duration with suicidal ideation across gender. After adjusting for potential confounders in the multivariable models, participants who cooked using solid fuel for long durations had the highest risks of suicidal ideation in the total population and across genders, the adjusted ORs (95% CIs) for suicidal ideation was 1.51 (1.22, 1.87) in total population, 1.92 (1.04, 3.42) in men and 1.46 (1.16, 1.85) in women, relative to clean fuel use with a short-duration cooking group. However, compared with those that cooked using clean fuel and for short durations, the significant associations between suicidal ideation and clean fuel use with long-duration or solid fuel use with short duration were not observed after adjusting for potential confounders.

### 3.3. Effect Modification of Kitchen Ventilation Condition on the Relationships of Domestic Cooking Fuel Type and Daily Cooking Duration with Suicidal Ideation

Table 3 shows the relationships between domestic cooking fuel type and daily duration of cooking duration with suicidal ideation according to kitchen ventilation conditions. In the natural ventilation group, the ORs (95% CIs) of solid fuel users versus clean fuel users and each 1 h increase in daily cooking duration for suicidal ideation were 1.33 (1.10, 1.60) and 1.24 (1.11, 1.38) in the multivariable-adjusted models, respectively. However, these significant relationships were not observed in those participants who cooked with mechanical ventilation apparatus in the kitchen. Similar results were observed in both genders.

**Figure 2:**
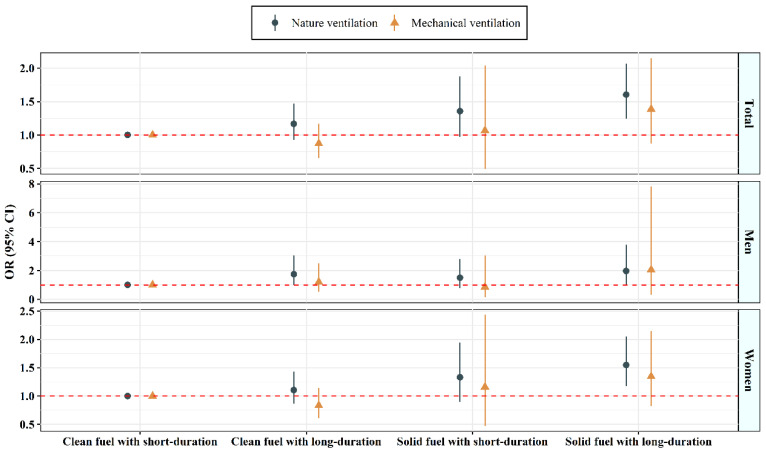
Combined association of fuel type and cooking duration with suicidal ideation stratified by ventilation.

Figure 2 illustrates the combined relationships of domestic cooking fuel type and dichotomous cooking duration with suicidal ideation according to kitchen ventilation conditions. In the natural ventilation group, the OR (95% CI) of using solid fuel for long-duration cooking versus using clean fuel for short-duration cooking was 1.60 (1.24, 2.07) after adjusting for potential confounders in the multivariable models. This positive association was also not observed in the mechanical ventilation group. Similar results were also observed in both genders.

Models adjusted for age, region, marital status, educational level, average monthly income, smoking status, drinking status, high fat diet, fruit and vegetable intake, physical activity, BMI, hypertension, T2DM, CHD, and stroke in men and women, and additionally adjusted for gender in total population.

## 4. Discussion

To our knowledge, this may be the first research to investigate the associations between HAP exposure and the risk of suicidal ideation in a rural region. The results indicated that HAP exposure from solid fuel use and long-duration cooking was associated with an increased risk of suicidal ideation However, these significant associations vanished in populations cooking with a mechanical ventilation instrument in the kitchen. These findings from this study have important implications for policymakers to reduce the risk of suicidal ideation through universal access to clean fuels and the installation of mechanical ventilation instruments in kitchens.

Despite the lack of research investing the relationship between HAP and suicidal ideation, the growing body of evidence has indicated consistently that exposure to ambient air pollution is positively associated with the risk of suicidal ideation [29,31,32], which may indirectly support the findings of this research. For instance, the findings of a prefectural panel study pointed to a positive correlation between the weekly concentration of PM_2.5_ exposure and suicidal ideation [31]. In addition, our previous research found that the ORs (95% CIs) for suicidal ideation associated with increases in NO_2_, PM_1_ and PM_2.5_ per 1 µg/m^3^ were 1.12 (1.04, 1.21), 1.08 (1.01, 1.15) and 1.10 (1.02, 1.19), respectively [26]. Another study reported that among the bereaved, each increase in the interquartile range (IQR) of PM_2.5_ was accompanied by a 10.55% (95% CI: 2.05%, 19.75%) increase in the risk of mortality by suicide for the first four days of mean exposure (mean lag of 0–3 days). [32]. Furthermore, ambient and domestic PM_2.5_ are mutually connected due to the air exchange between ambient and household air [33], with PM_2.5_ generated from domestic cooking activity constituting a critical part of ambient PM_2.5_ [34]. As aforementioned evidence, the increased risk of suicidal ideation attributable to HAP may be explained by the increased concentration of domestic PM_2.5_.

Domestic cooking fuel type, daily duration of cooking and kitchen ventilation conditions may influence the concentration of HAP such as domestic PM_2.5_ and further influence the risk of suicidal ideation. One study has illustrated that the concentration of domestic PM_2.5_ produced by using solid fuels during cooking can be several orders of magnitude higher compared with those cooked with electricity or gas [35,36]. In addition, personal exposure to PM_2.5_ concentrations was significantly associated with the duration of daily cooking activity [37,38]. A cross-sectional study conducted in Ethiopia showed a positive association between self-reported cooking duration and 24-h average measurement of PM_2.5_ (µg/m^3^), with a correlation coefficient of 0.49 and *p*-value of 0.001 [38]. Mechanical ventilation equipment used in the kitchen may attenuate solid fuel use and long-duration cooking-induced suicidal ideation risk by reducing the concentration of domestic PM_2.5_ generated by cooking [39,40,41]. Evidence indicated that the efficiency of the range hood in removing PM_2.5_ was 58% ± 6% during a typical Chinese Residential Cooking [42]. Taken together, propelling clean fuels and installing mechanical ventilation instruments in the kitchen should be taken into consideration to reduce the concentration of domestic air pollutants and further reduce the HAP-related public health burden.

Whilst the biological mechanisms underlying the relationship of HAP with domestic solid fuel use, long-duration cooking and poor kitchen ventilation condition with suicidal ideation were yet not well established, several potential explanations should be observed. First, gases and PM inhaled in households can give rise to inflammation and oxidative stress, and that in turn plays a pivotal function in the progression of suicidal ideation [28,43,44]. Second, PM was reported to increase the risk of suicidal ideation through over-activating the hypothalamic–pituitary–adrenal axis and altering the activity of serotonergic neurotransmission [45,46]. Third, the neuroplasticity and structural impairment of the hippocampus induced by PM may further lead to depression-like responses and cognitive decline, which has been proven to be associated with suicidal ideation [47,48,49]. Finally, exposure to HAP may increase the risk of a lot of chronic diseases, such as stroke [50], and chronic obstructive pulmonary disease [51], and these diseases may in turn increase the risk of suicidal ideation [52,53].

Several limitations in this study should be taken into account. First, given that this research was cross-sectional, the causality of domestic cooking fuel type, daily duration of cooking activity and kitchen ventilation condition with suicidal ideation could not be determined. However, a follow-up investigation of this cohort study is ongoing. Data from prospective studies will be available to further explore the relationship between HAP and suicidal ideation. Second, multi-stage stratified cluster sampling may lead to sampling bias. However, the sample size of this study is large and the five regions where the study was conducted are evenly distributed in the eastern, western, northern, southern and central parts of Henan Province. Third, using cooking fuel type and cooking duration as proxy indicators of HAP rather than direct measurement of air pollution parameters may lead to potential bias. However, solid fuel use is commonly used to reflect HAP in large epidemiologic studies and research aimed at directly measuring HAP indicators is underway. Fourth, item 9 of the PHQ-9 may not screen for suicidal ideation cases as accurately as other instruments such as the Beck Suicide Inventory. However, the PHQ-9 is in accordance with the Diagnostic and Statistical Manual for Mental Disorders criteria and previous studies have demonstrated the applicability of this method [28,30]. Fifth, HAP was assessed by self-reported questionnaires rather than direct measurement in this research, which may lead to exposure misclassification and recall bias. Nevertheless, a previous study showed a kappa coefficient of 0.61 between the baseline survey and re-survey [54], implying a high degree of agreement. Sixth, environmental noise may also be a potential confounding factor that may have a non-negligible effect between HAP and suicidal ideation [55]. However, this research was conducted in the rural areas of Henan province where the environment is relatively quiet. Seventh, the study was carried out in rural Henan Province and the results may not be generalisable to other areas, but solid fuels are mainly used in these economically disadvantaged areas. Finally, although multiple confounders have been controlled for, some of the other covariates such as the type of heating fuel and kitchen location in the households in association with HAP and suicidal ideation were not taken into consideration.

## 5. Conclusions

The findings from this study indicate that domestic solid fuel use and daily long-duration cooking were related to an increased risk of suicidal ideation, and these positive relationships could be ameliorated in participants cooking with mechanical ventilation instruments in the kitchen in rural areas. These results support that the use of mechanical ventilation instruments in the kitchen is an effective measure to reduce HAP-associated suicidal ideation risk, emphasizing the importance of HAP control in resource-limited areas.

## Figures and Tables

**Table 1:** Basic characteristics of study participants according to suicidal ideation (*n* = 21381).

Variables	Total	Non-SI	SI	*p*
	(*n* = 21,381)	(*n* = 20,557)	(*n* = 824)	
**Age (years, mean ± SD)**	55.37 ± 11.83	55.30 ± 11.84	57.29 ± 11.36	<0.001 ^a^
**Gender (*n*, %)**				<0.001 ^b^
Men	4872 (22.79)	4740 (23.06)	132 (16.02)	
Women	16,509 (77.21)	15,817 (76.94)	692 (83.98)	
**Marital status (*n*, %)**				<0.001 ^b^
Married/cohabiting	19,009 (88.91)	18,319 (89.11)	690 (83.74)	
Widowed/divorced/separated/single	2373 (11.09)	2238 (10.89)	134 (16.26)	
**Educational level (*n*, %)**				<0.001 ^b^
Elementary school or below	10,316 (48.25)	9810 (47.72)	506 (61.41)	
Junior high school	7877 (36.84)	7638 (37.16)	239 (29.00)	
Senior high school or above	3188 (14.91)	3109 (15.12)	79 (9.59)	
**Average monthly income (*n*, %)**				<0.001 ^b^
<500 RMB	7639 (35.73)	7236 (35.20)	403 (48.91)	
500–1000 RMB	11,848 (55.41)	11,480 (55.84)	368 (44.66)	
≥1000 RMB	1894 (8.86)	1841 (8.96)	53 (6.43)	
**Smoking status (*n*, %)**				<0.001 ^b^
Never	18,030 (84.32)	17,290 (84.11)	740 (89.81)	
Former	979 (4.58)	955 (4.65)	24 (2.91)	
Current	2372 (11.09)	2312 (11.25)	60 (7.28)	
**Drinking status (*n*, %)**				<0.001 ^b^
Never	18,472 (86.39)	17,721 (86.20)	751 (91.14)	
Former	599 (2.80)	587 (2.86)	12 (1.46)	
Current	2310 (10.80)	2249 (10.94)	61 (7.40)	
**High-fat diet (*n*, %)**	3497 (16.36)	3390 (16.49)	107 (12.99)	0.008 ^b^
**High fruit and vegetable intake (*n*, %)**	10,314 (48.24)	9868 (48.01)	446 (54.13)	0.001 ^b^
**Physical activity (*n*, %)**				0.118 ^b^
Low	6270 (29.33)	6054 (29.45)	216 (26.21)	
Moderate	8671 (40.55)	8316 (40.45)	355 (43.08)	
High	6440 (30.12)	6187 (30.10)	253 (30.70)	
**BMI (kg/m^2^, mean ± SD)**	24.80 ± 3.77	24.83 ± 3.76	24.16 ± 3.79	<0.001 ^a^
**Hypertension (*n*, %)**	6939 (32.43)	6674 (32.47)	261 (31.67)	0.634 ^b^
**Stroke (*n*, %)**	1395 (6.52)	1285 (6.25)	110 (13.35)	<0.001 ^b^
**CHD (*n*, %)**	1186 (5.55)	1093 (5.32)	93 (11.29)	<0.001 ^b^
**T2DM (*n*, %)**	1893 (8.85)	1792 (8.72)	101 (12.26)	<0.001 ^b^
**Fuel type (*n*, %)**				<0.001 ^b^
Clean fuel	17,232 (80.59)	16,660 (81.04)	572 (69.42)	
Solid fuel	4149 (19.41)	3897 (18.96)	252 (30.58)	
**Cooking duration (h/day, mean ± SD)**	1.42 ± 0.82	1.41 ± 0.82	1.57 ± 0.87	<0.001 ^a^
**Ventilation (*n*, %)**				<0.001 ^b^
Natural ventilation	10,976 (51.34)	10,414 (50.66)	562 (68.20)	
Mechanical ventilation	10,405 (48.66)	10,143 (49.34)	262 (31.80)	

Abbreviation: SD, standard deviation; RMB, Renminbi; BMI, body mass index; CHD, coronary heart disease; SI, suicidal ideation. ^a^ *t*-test was performed to compare the differences in continuous variables; ^b^ Chi-square test was used to compare the differences in the categorical variables.

**Table 2:** Associations of fuel type and cooking duration with suicidal ideation.

Models	Total	Men	Women
	(*n* = 21,381)	(*n* = 4872)	(*n* = 16,509)
**Model 1**			
**Fuel type**			
Clean fuel	1.00	1.00	1.00
Solid fuel	1.88 (1.62, 2.19)	1.83 (1.23, 2.68)	1.87 (1.58, 2.21)
**Cooking duration (h/day)**	1.25 (1.16, 1.36)	1.39 (1.13, 1.69)	1.17 (1.06, 1.28)
**Model 2**			
**Fuel type**			
Clean fuel	1.00	1.00	1.00
Solid fuel	1.67 (1.42, 1.95)	1.71 (1.13, 2.56)	1.63 (1.37, 1.93)
**Cooking duration (h/day)**	1.23 (1.13, 1.34)	1.34 (1.08, 1.65)	1.15 (1.04, 1.26)
**Model 3**			
**Fuel type**			
Clean fuel	1.00	1.00	1.00
Solid fuel	1.37 (1.16, 1.62)	1.30 (0.84, 1.99)	1.39 (1.16, 1.67)
**Cooking duration (h/day)**	1.15 (1.05, 1.26)	1.26 (1.00, 1.56)	1.14 (1.03, 1.26)

Model 1 was unadjusted; Model 2 adjusted for age and region in men and women, and additionally adjusted for gender in the total population; Model 3 further adjusted for marital status, educational level, average monthly income, smoking status, drinking status, high fat diet, fruit and vegetable intake, physical activity, BMI, hypertension, T2DM, CHD, stroke, ventilation and fuel type or cooking duration based on model 2.

**Table 3:** Associations of fuel type and cooking duration with suicidal ideation stratified by ventilation.

Models	Total	Men	Women
	(*n* = 21,381)	(*n* = 4872)	(*n* = 16,509)
**Natural ventilation**			
**Fuel type**			
Clean fuel	1.00	1.00	1.00
Solid fuel	1.33 (1.10, 1.60)	1.27 (0.78, 2.06)	1.34 (1.09, 1.64)
**Cooking duration (h/day)**	1.24 (1.11, 1.38)	1.43 (1.08, 1.86)	1.22 (1.08, 1.37)
**Mechanical ventilation**			
**Fuel type**			
Clean fuel	1.00	1.00	1.00
Solid fuel	1.40 (0.96, 1.99)	1.14 (0.33, 3.03)	1.46 (0.97, 2.13)
**Cooking duration (h/day)**	1.03 (0.88, 1.21)	0.96 (0.59, 1.44)	1.04 (0.87, 1.23)

Models adjusted for age, region, marital status, educational level, average monthly income, smoking status, drinking status, high fat diet, fruit and vegetable intake, physical activity, BMI, hypertension, T2DM, CHD, stroke, either fuel type or cooking duration in men and women, and additionally adjusted for gender in total population.

## Data Availability

The data analyzed during the current study are available from the corresponding author upon reasonable request.

## Supplementary Materials

The following supporting information can be downloaded at: https://www.mdpi.com/article/10.3390/toxics10120721/s1. The detailed calculations of the sample size are shown.
