# Supplementary material for: The Effect of Kitchen Ventilation Modification on Independent and Combined Associations of Cooking Fuel Type and Cooking Duration with Suicidal Ideation: A Cross-Sectional Study
Source: Toxics. 2022 Nov 24;10(12):721. doi: 10.3390/toxics10120721 (PMC9788604; doi:10.3390/toxics10120721)
Supplement: Supplementary file 1 [file toxics-10-00721-s001.zip › toxics-2038133-supplementary.pdf]

The sample size of this study was calculated as follows:

Groups	Risk factor		Total
	Exposed group	Non-exposed group	
Case Group	a	b	n_1
Control group	c	d	n_2

$$\pi_1 = a/n_1$$

$$\pi_2 = c/n_2$$

$$Var\left(In\widehat{OR}\right) = \frac{1}{a} + \frac{1}{b} + \frac{1}{c} + \frac{1}{d}$$

$$In(1-\varepsilon) = -Z_{\alpha/2}\sqrt{Var(In\widehat{OR})}$$

$$n = \frac{Z_{\alpha/2}^2\{1/[\pi_1(1-\pi_1)] + 1/[\pi_2(1-\pi_2)]\}}{[In(1-\varepsilon)]^2}$$

$$\alpha = 0.05$$

According to the calculation of the above formula, the sample size required for this study is 1860. The sample size for our study is 21381, which is far larger than the required sample size.
